# Modeling and simulation of belt bucket elevator head shaft for safe life operation

**DOI:** 10.1038/s41598-022-26060-x

**Published:** 2023-01-19

**Authors:** Peter Okechukwu Chikelu, Solomon Chuka Nwigbo, Obotowo William Obot, Paul Chukwulozie Okolie, Jeremiah L. Chukwuneke

**Affiliations:** 1 Department of Mechanical Engineering, Nnamdi Azikiwe University, Awka, Anambra State Nigeria; 2 Department of Mechanical and Aerospace Engineering, University of Uyo, Uyo, Akwa Ibom State Nigeria

**Keywords:** Mechanical engineering, Engineering

## Abstract

This research paper presents a step by step conceptual design and life prediction approach for the design, modeling and simulation of head shaft of a belt bucket elevator, to be used for conveying grains to a height of 33.5 m and at the rate of 200 tons/h. output. For this elevator system, the force and torque acting on the head shaft as well as the bending moment were calculated. Furthermore, the diameter of each cross section of the shaft was determined taking into consideration the geometric and fatigue stress concentration factors, due to shoulders which contribute significantly to most fatigue failures of shafts. The stress induced on the shaft by the force and the factor of safety for each cross section of the shaft was calculated using the DE-Goodman criterion. The model of the shaft was created from the calculated diameters and subjected to static and fatigue analysis using SolidWorks FEA. The results were validated by comparing the values from the FEA and the calculated values for stress and factor of safety of the critical section of the shaft, which showed an equivalent value. The FEA gave a fatigue load factor greater than one, which signifies that the shaft will not go into failure mode within the infinite life cycle of the shaft. The value of the fatigue strength obtained from FEA was higher than the value for the maximum von misses stress of the shaft, this result shows that the head shaft will sustain the loading stresses over a finite life prediction. This research is significant because the stress induced forces on the head shaft from each component of the elevator system were properly identified and analyzed so as to obtain precise results for life prediction.

## Introduction

**Figure 1:**
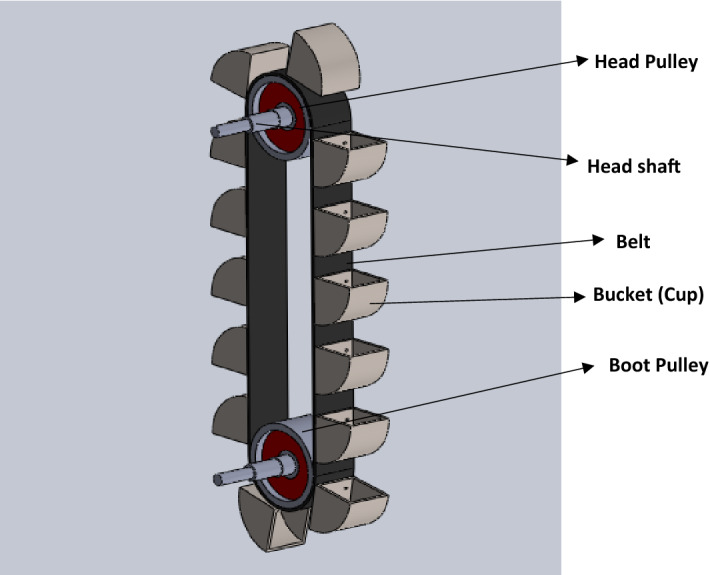
Bucket belt elevator system.

Grains such as corn and wheat produced from farms and processed in industry are first stored in silo facility to ensure all year round availability of food for human consumption. These grains are usually discharged into the silo by means of a belt bucket elevator system, which is one of the important material handling equipment used in industries to convey bulk materials from the ground level to a required height. The belt bucket elevator system (Fig. 1) consists of a flat flexible belt with cup-shaped buckets attached to it. The belt is firmly joined over a spaced head and boot pulleys and driven by the head pulley. During the rotational drive, the buckets are filled with grains at the elevator boot (bottom), conveyed to the top where the grains are discharged as the bucket turns downward over the head pulley. The head pulley is attached to the head shaft which is rotated by a drive mechanism (Gearbox motor). Generally, a shaft is a component of a mechanical device usually of circular cross section which transmits motion or power from one point to another. It is usually stepped to provide shoulder for other mechanical components. The head shaft of a belt bucket elevator system is an important structural component of the elevator system because it is the drive shaft of the system which supports the weight of the belt, the weight of buckets filled with grains and the head pulley, hence any failure of the head shaft means a failure of the entire elevator system^1,2^. A broken head shaft can be devastating with consequences such as loss production time with its associated downtime cost, drop in quality of grains and wastage (due to moisture and weevils’ infestation). Also of safety concern, the procedure for replacement of the failed shaft is very difficult, most especially for very tall and heavy elevators, where the belts and cups will have to be suspended to remove the fractured shaft, this poses a significant safety risk to the maintenance engineers. Thus considering the load, the shaft will be subjected to and the consequences of any failure, a proper design of the head shaft of the elevator is critical and a generous safety factor for an infinite life is recommended to ensure safe and reliable operation of a developed elevator system^3,4^. The head shaft should be designed to sustain both static and cyclic loading in its service life. The cyclic load carried by the shaft causes bending which produces stresses within the shaft and results in fatigue failure when the cyclic loading over a period of time leads to the failure of the shaft even when the magnitude of the stress is below the material yield strength. Since it is on record that majority of engineering shaft failures are caused by fatigue, it is recommended that at the conceptual design stage, shafts should be correctly sized to prevent fatigue failure within the expected period of usage by the industry^5^.

There are many literature which have dealt with problems relating to elevator shaft system. Goksenli et al.^6^ investigated the failure of an elevator drive shaft due to torsion-bending fatigue and found out that failure occurred at the keyway of the shaft due to faulty design of the keyway, which resulted to a high notch effect at the keyway section. Brijesh et al. optimized the weight and discharge capacity as well as the stress and deformation of the bucket and shaft of a modeled belt driven elevator for material transport using SolidWorks^7^. Chavhan et al.^8^ studied the load carrying capacity of an elevator bucket, the strain in the bucket edges and the clamp bolts using CATIA V5 3D model and ANSYS software and concluded that failure of elevator shaft was the main cause of the bucket failure. Gililland et al. reported that the loads imposed on the boot shaft are very low when compared to the head shaft. This establishes the critical nature of the head shaft of an elevator system^9^. Snehal et al.^10^ reviewed that failure on shaft takes place due to high stress on the keyway and areas where there are abrupt changes in cross sectional area. Yin et al.^11^ investigated the failure of a bucket elevator chain link and reported that the failure was due to propagation of crack embedded within the material during loading. This signifies that the elevator shaft was equally affected by such loading. The drive shaft of a system was examined and reported to have failed under fatigue due to design error of the fillet radius of the shaft^12^. Ariwibowo et al.^13^ equally investigated the failure of a shaft and reported that the failure was due to crack propagation initiated from fatigue loading.

Osakue developed a reliable model for a probabilistic design approach for shafts under combined bending and torsional loading, it also reported that for conditions of combined torsional and bending stress, distortion energy (DE) theory of failure was appropriate for the design analysis of the component^14^. Baig et al.^15^ investigated a shaft for failure and reported that failure always occurs at the areas of high stress concentration. Pelaseyed et al.^16^ studied the failure of the shaft of a unit and concluded that the failure was as a result of fatigue loading. Gurudath et al.^17^ investigated the failure of a bucket elevator shaft and reported that the failure was due to crack initiated at the heat affected zone of tack-welded spot on the shaft which propagated transversely by fatigue due to cyclic loading. Butlovic et al.^18^ study on design of machine parts such as shaft inferred that the use of CAD softwares provides simple and exact values of stress and deformation distribution on the shaft. It was also reported that the Goodman theory was widely used and adjudged to be a safe design approach in case of combined static and cyclic stress. Adekunle et al.^19^ developed a software for shaft design that satisfactorily handled design based on strength and safety. Sathishkumar et al.^20^ created a shaft model using CREO software, performed static analysis of the model to determine the stress and deformation under load using ANSYS software, it was concluded that the result obtained in good agreement and is within the safe limit. Ofolabi et al. investigated the parameters of fatigue life of a shaft and produced a 3D model of the shaft using Inventor software. It was reported that the results of the finite element analysis when compared with calculate values were satisfactory^21^. Engel et al. studied the failure mode of a shaft, analytic method was carried out to determine the stress and deformation of the shaft under combined torsion and bending loads, the results where then compared with that of finite element analysis method. It was concluded that the shaft needs some surface treatment to increase its fatigue life^22^. Rasovic et al. analyzed a SolidWorks-modeled drive shaft using finite element analysis and reported unsatisfactory result from the analysis as a result of the shaft geometrical change and low yield strength of the shaft material^23^. Robothan et al.^24^ analyzed a shaft and concluded that the behavior of shaft under combined loads can be accurately predicted using finite element analysis.

With the level of advancement in mechanical design in this era, shaft prototypes with optimal performance are developed through modeling of the physical system, as this helps to eliminate failure which comes with costly consequences^25,26^. With model-based design approach, shaft design engineers can make accurate prediction of the system performance as well as its service life, and make corrections of potential failure points of the shaft at the conceptual design stage. In this study, the limitations to obtaining safe shaft design for infinite life, such as the presence of stress risers and the centrifugal force action were analyzed using standard shaft design calculation procedure. The model of the head shaft was produced and simulated, so as to make accurate prediction of the service life of the designed head shaft.

Hence, this research work provides a comprehensive approach for design and modeling of the head shaft of a belt bucket elevator system which will not fail under fatigue as well as in predicting the service life of the shaft, using DE-Goodman criterion and SolidWorks engineering software.

## Methodology

### Physical model

**Figure 2:**
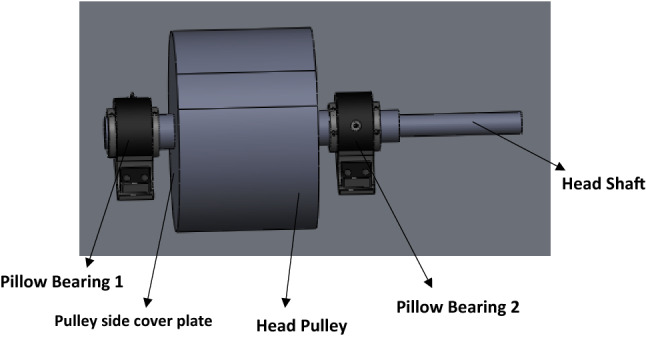
Mechanical model of the elevator head shaft with pulley and its bearing support.

The assembly model of the bucket elevator head as shown in Fig. 2 consists of the head pulley coupled to a head shaft supported by two self-aligning pillow bearings. The dimensions for the design analysis of the pulley were chosen at the conceptual design stage while that of the shaft were derived from the design calculations.

### Material selection

For the design, AISI 1020 steel was selected for the pulley because of its high ductility, machinability, strength, polished finished property and good weldability. AISI 1018 steel was selected for the elevator cups because of it great ductility, malleability, toughness, ease of welding and cheaper cost. AISI 1045 cold drawn steel was selected for the shaft because of its high tensile strength, hardness, excellent size accuracy, straightness and good surface condition^27–29^.

### Design methodology

**Figure 3:**
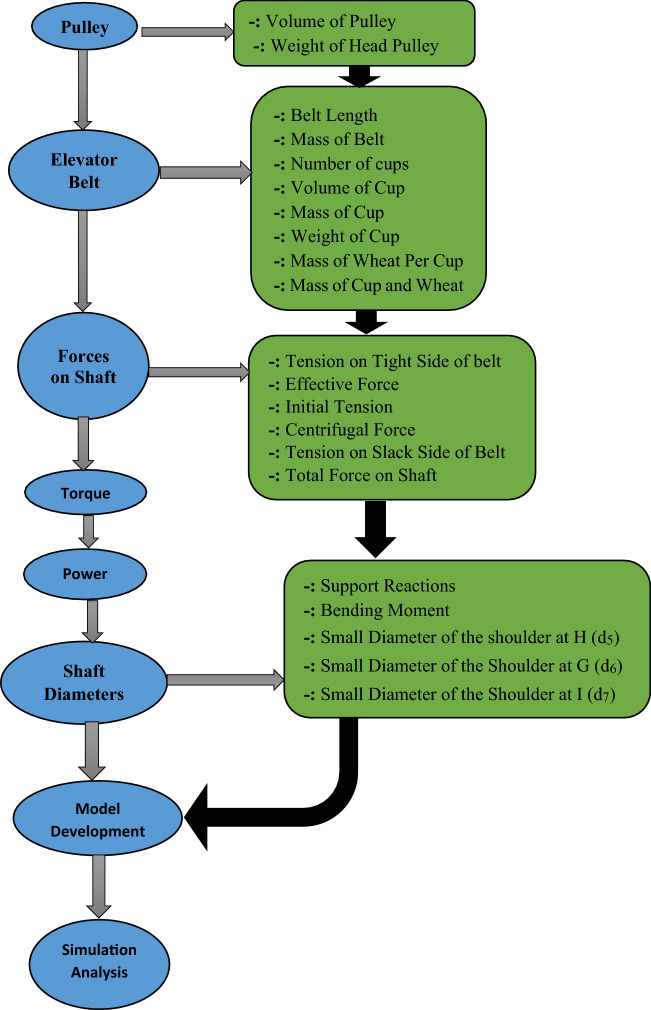
Graphical representation of the methodology.

During operation of the belt bucket elevator system, the head shaft is subjected to torque from the gear drive motor and bending moment from the weight of the belt, cups filled with wheat grains and pulley, these generates torsional shear stress and bending stresses on the shaft, because of these reasons, the shaft was designed for combined stress using distortion energy theory of failure. The shaft design involves the determination of the preliminary diameter of the shaft. The graphical representation of the methodology is shown in Fig. 3.

#### Hypothesis

For the model based design, it was considered that there was no resisting friction from the bearing and the weight of the shaft was negligible. It was also considered that both the pulley and the gear drive were mounted to the shaft using shrink disc, hence, the need for welding and keyway which act as stress risers was eliminated. It was also considered that the gear drive was well suspended by external clamp, hence its weight was not on the shaft. The grain product used was wheat.

The design analysis covers some areas which includes:

### Pulley

#### Volume of pulley (Vp)

**Figure 4:**
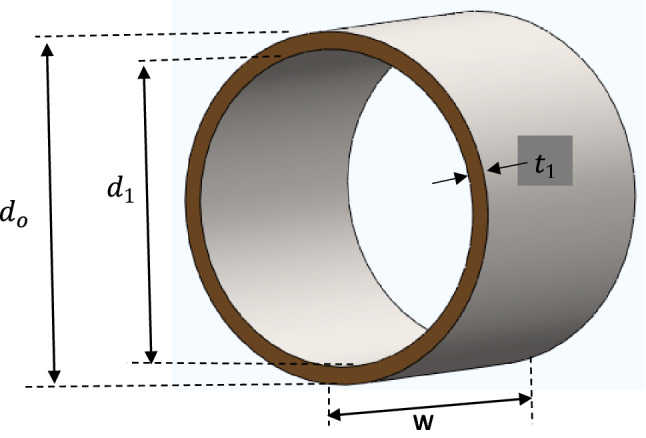
Hollow cylinder of the Pulley.

**Figure 5:**
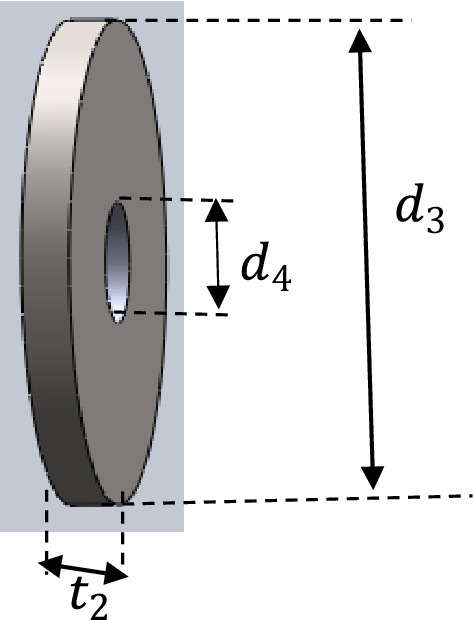
Side cover plates of pulley.

The head pulley of the elevator is a crowned type which uses flat belt***. ***To determine the volume of the pulley, we split the pulley into two sections as shown in Figs. 4 and 5.

The hollow cylinder and the two side plates of the pulley were considered. The volume of the pulley was determined using Eq. (1)

1 $$ V_{p} = \frac{\pi W}{4}\left[ {d_{o}^{2} - d_{1}^{2} } \right] + \frac{{\pi t_{2} }}{2}\left[ {d_{3}^{2} - d_{4}^{2} } \right] $$

where V_P—_Volume of pulley, W—Width of pulley, d_O_—External diameter of pulley(drum), d_1—_Internal diameter of pulley, d_3_—External diameter of pulley side cover plate, d_4—_Internal diameter of pulley side cover plate, t_1_—Thickness of pulley plate, t_2_—Thickness of side cover plate.

#### Weight of head pulley

The weight(N) of the head pulley was determined from Eq. (2),

2 $$ W_{P} = \rho \; \times \; V_{P} \; \times \; g $$

where W_P_—Weight of the head pulley, $\rho$—Mass density of pulley material, g—Acceleration due to gravity.

### Elevator belt

#### Belt length

**Figure 6:**
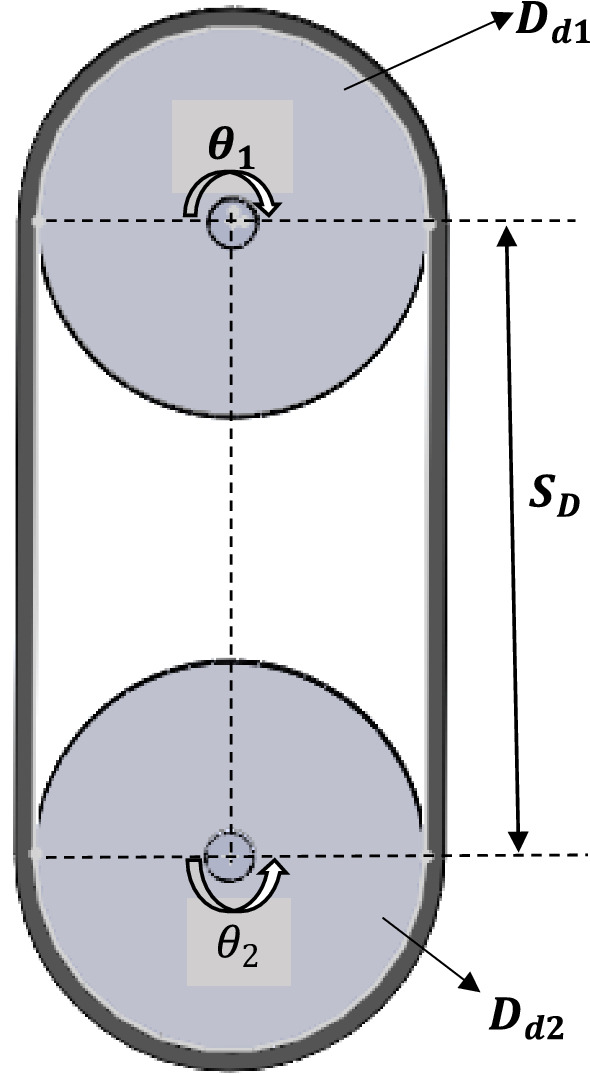
Sketch diagram of the belt pulley arrangement in belt elevator system.

The elevator operates on an open belt drive system as shown in Fig. 6.

The length of the open belt drive was determined from Eq. (3) given as

3 $$ L_{B} = \frac{\pi }{2}\left[ {D_{d1} + D_{d2} } \right] + \left[ {\frac{{(D_{d1} - D_{d2} )^{2} }}{{4 \; \times \; S_{D} }}} \right] + 2 \; \times \;S_{D} $$

where L_B_—length of belt, D_d1_—Diameter of the head pulley (Drive pulley), D_d2_—Diameter of the bottom pulley (Driven pulley), S_D_—Centre to centre shaft distance,

*θ*_1_, *θ*_2_—Angel of contact (or wrap) for the head and bottom pulleys successively^30,31^.

#### Mass of belt (M_B_)

The mass of the elevator belt was determined from Eq. (4) given as

4 $$ M_{B} = \rho_{B} \; \times \; W_{tB} \; \times \; t_{B} \; \times \; L_{B} $$

where $\rho_{B}$—Density of belt, W_tB_—Width of the belt, t_B_—Thickness of belt, L_B_—Length of Belt.

The density of the belt was also determined from Eq. (5) as:

5 $$ \rho_{B} = M_{BL} \left( { \frac{1}{{W_{tB} \; \times \; t_{B} }}} \right) $$

where M_BL_—mass of belt per metre length.

Furthermore, the weight of the belt (W_B_) was determined using Eq. (6)

6 $$ W_{B} = M_{B} x g $$

#### Number of cups (T_NC_)

The total number of cups on the elevator was determined from Eq. (7) given as;

7 $$ T_{NC} = N_{CL} x L_{B} $$

where **N**_**CL**_—Number of cups per metre.

#### Volume of cup (Wc)

**Figure 7:**
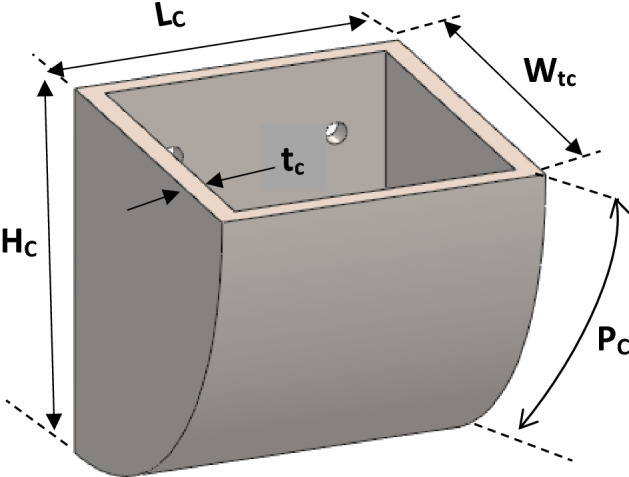
Sketch diagram of elevator cup (bucket).

The elevator cup was sketched as represented in Fig. 7.

**Figure 8:**
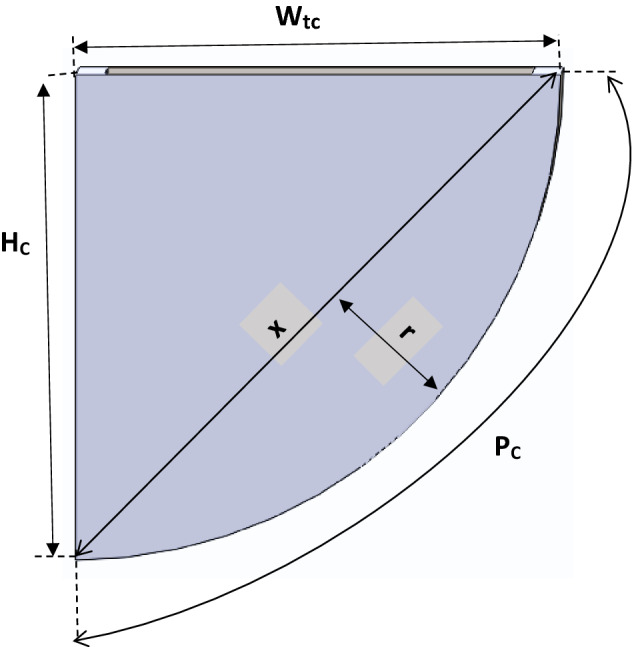
Section of the elevator cup (bucket).

From the sketched diagram, the volume of the cup was determined mathematically by considering a section of the cup in Fig. 7 and representing it in Fig. 8 as a triangle and semi-ellipse joined as a section.

From Fig. 8, the length of X was determined from the triangular section using Pythagoras theorem and this was given in Eq. (8) as:

8 $$ X = \sqrt {H_{C}^{2} + W_{C}^{2} } $$

where X—Hypotenuse of the triangle, H_C_—Height of the cup, W_C_—Width of the cup.

Similarly, the radius(r) of the semi-ellipse section of Fig. 8 was determined using the derived Eq. (9) given below;

9 $$ r = \frac{{4P_{eC} - X\pi }}{2\pi } $$

where r—radius of semi- ellipse, P_C_ ≈ P_ec_—Perimeter of semi-ellipse (surface curve length).

Thus, considering the hollow section of the elevator cup, the volume of the cup (V_C_) was determined as the difference between the volume of external cup dimension and the volume of the internal cup dimension; this is equivalent to the difference of the sum of the area of the triangle and area of the semi-ellipse multiplied by length for external section and that of the sum of the area of the triangle and area of semi-ellipse multiply by length for the internal section of the cup. Thus, the volume of the cup material was determined using derived Eq. (10) as given:

10 $$ V_{C} = \left[ {\left( {\frac{{W_{C} H_{C} }}{2} + \frac{\pi Xr}{4}} \right)L_{C} } \right] - \left[ {\left( {\frac{{(W_{C} - 2t_{C } )\left( {H_{C} - t_{C} } \right) }}{2} + \frac{\pi Xr}{4}} \right)\left( {L - 2t_{c} } \right)} \right] $$

where L_C_—Length of the cup, W_tc_—Width (or projection) of cup, t_c_—Thickness (gauge) of cup material.

#### Mass of cup (M_C_)

The mass of each elevator cup was determined using Eq. (11) as shown below,

11 $$ M_{C} = \rho_{C} \; \times \;V_{C} $$

where $\rho_{C}$—Mass density of elevator cup material, V_C_—Volume of elevator cup.

#### Weight of cup (Wc)

The weight of each elevator cup was determined using Eq. (12) given below^32^,

12 $$ W_{C} = M_{C} \; \times \; g $$

#### Mass of wheat per cup (M_W_)

It is worthy to note that during operation, only 67% of the designed cup capacity is actually filled with the grain(wheat). Thus, the actual mass of wheat (excluding the cup weight) only transported by each cup was given in Eq. (13) as:

13 $$ M_{W} = \rho_{g} \; \times \;C_{C} \; \times \; 0.67 $$

where ${\uprho }_{{\text{g}}}$—Bulk density of grain (wheat)**,** $C_{C}$—Designed capacity of elevator cup.

The designed capacity of the elevator cup is equivalent to the volume of internal cup dimension, hence, the designed capacity of the elevator cup was determined from Eq. (14)^33,34^

14 $$ C_{C} = \left( {\frac{{W_{C} - 2t_{C} }}{2}} \right)\left( {H_{C} - t_{C} } \right) + \left( {\frac{\pi Xr}{4}} \right)\left( {L_{C} - 2t_{C} } \right) $$

#### Mass of cup and wheat (M_CW_)

The mass of each elevator cup carrying wheat was determined using Eq. (15)

15 $$ M_{CW} = M_{C} + M_{W} $$

### Forces on shaft (F_S_)

**Figure 9:**
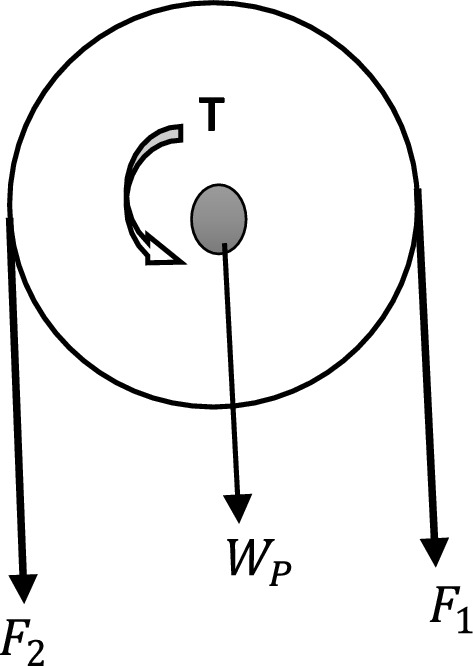
Free body diagram (FBD) of top section of belt elevator system.

The forces acting on the shaft are the weights of the head pulley, elevator cups, grain (wheat) and the belt. This was resolved using the free body diagram shown in Fig. 9

Where F_1_—Tension(force) on tight side of belt, F_2_—Tension(force) on the slack side of belt, W_P_—weight of the head pulley, T—Torque.

#### Tension on tight side of belt (F_1_)

Taking centrifugal force into consideration, the force/tension on the tight side (F_1_) of the belt was determined using Eq. (16) below:

16 $$ F_{1} = F_{P} + F_{ce} + \frac{{F_{C} }}{2} $$

where F_P_—Initial (Preload) tension(force), F_CE_—Tension due to centrifugal force,

F_C_- Effective/circumferential force.

#### Effective/circumferential force (F_C_)

For the elevator belt to rotate, the circumferential force must be equal to the frictional force (F_F_) of the belt on the pulley interface (i.e. F_F_ ≈ F_C_).

For this elevator system, the frictional force (F_F_) was given in Eq. (17) as:

17 $$ F_{C} \approx F_{F} = \mu .g\left[ {M_{B} + \frac{{M_{CW } \; \times \;T_{NC} }}{2} + \frac{{M_{C } \; \times \;T_{NC} }}{2}} \right] $$

where $\mu$—Coefficient of friction of elevator belt.

The coefficient of friction $\left( \mu \right)$ of the elevator belt was determined from Eq. (18)

18 $$ \mu = 0.54 - \left( { \frac{42.6}{{152.6 + V_{eb} }}} \right) $$

where V_eb_—Velocity of the belt.

Considering the design capacity of the elevator, the velocity of the belt was determined from Eq. (19)

19 $$ V_{eb} = \frac{Q}{{M_{W } \; \times \; N_{CL} \; \times \; 3600}} $$

where Q—Capacity of the bucket elevator.

To ensure an effective throw of the grain(wheat) into the chute at the head pulley, a speed in the range of 1–2 m/s is recommended for centrifugal bucket elevator. Also, it has been reported that rubber coated flat belts running over pulleys in practice displays friction coefficient between 0.3 and 0.8. Thus, these criteria were used as validation values during the analysis^35–37^.

#### Initial tension (F_P_)

The Initial tension or preload force of the belt on the shaft was determined using Eq. (20)

20 $$ F_{P} = F_{C} \left[ {\frac{{\left( {e^{{\mu \theta_{1} }} } \right) + 1}}{{2\left( {(e^{{\mu \theta_{1} }} ) - 1} \right)}}} \right] $$

The angle of contact for the head (θ_1_) in radians was determined from Eq. (21) given as^38^:

21 $$ \theta_{1} = \left[ {180 + 2 \; \times \; Sin\left( {\frac{{D_{d1} - D_{d2} }}{{2 x S_{D} }}} \right)} \right]\; \times \; \frac{\pi }{180} $$

#### Centrifugal force (F_ce_)

For the elevator system, centrifugal forces are generated at the angle of wrap where the belt rotates around the pulley. The centrifugal force (F_ce_) was determined from the derived Eq. (22)

22 $$ F_{ce} = \left[ {\frac{{\left( {M_{CW} \; \times \; {\raise0.7ex\hbox{${N_{CA} }$} \!\mathord{\left/ {\vphantom {{N_{CA} } 2}}\right.\kern-\nulldelimiterspace} \!\lower0.7ex\hbox{$2$}}} \right) + \left( {M_{C} \; \times \; {\raise0.7ex\hbox{${N_{CA} }$} \!\mathord{\left/ {\vphantom {{N_{CA} } 2}}\right.\kern-\nulldelimiterspace} \!\lower0.7ex\hbox{$2$}}} \right)}}{{L_{ce} }}} \right]V_{eb}^{2} $$

where L_ce_—Length of belt contact on the pulley with reference to the angle of wrap ≈ Arc length of the pulley, N_CA_—Number of elevator cups within the Arc length of the pulley.

**Figure 10:**
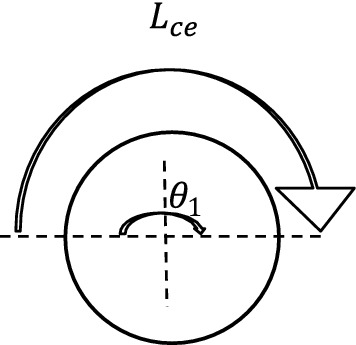
Diagram of head pulley/belt contact.

Furthermore, the elevator was designed such that the diameters of the head and bottom pulleys are equal, for this reason, the length of the belt contact on the pulley with reference to the angle of wrap will be equal to the length of semi-circle of the head pulley, this is shown in Fig. 10.

Therefore, Length of belt contact on the pulley with reference to the angle of wrap was determined from the derived Eq. (23)

23 $$ L_{ce} = \left( \pi \right)\; \times \;\frac{{D_{d1} }}{2} $$

The Number of cups within the arc length (N_CA_) was determined from the Eq. (24)

24 $$ N_{CA} = N_{CL} \; \times \;L_{ce} $$

#### Tension on slack side of belt (F_2_)

The tension on the slack side of the elevator belt was determined using Euler model in Eq. (25), which is the simplest theoretical model for belt drives^39,40^.

25 $$ \frac{{F_{1} }}{{F_{2} }} = e^{{\mu \theta_{1} }} $$

#### Total force on shaft (F_S_)

The total force acting of the shaft was determined from Eq. (26) stated below:

26 $$ F_{s} = F_{1} + F_{2} + W_{P} $$

#### Torque (T)

The total torque(T) required to operate the elevator system was determined from Eq. (27) given as T = Torque from belt (T_B_) + Torque to rotate the head pulley (T_HP_) + Torque to rotate the bottom pulley (T_BP_).

This is represented thus:

27 $$ T = \frac{{\left( {F_{1} - F_{2} } \right)D_{d1} }}{2} + J_{1} \alpha_{1} + J_{2} \alpha_{2} $$

where J_1_—Mass moment of Inertia of the head pulley, J_2_—Mass moment of Inertia of the bottom pulley, $\alpha_{1} - \user2{ }$ Angular acceleration of the head pulley, $\alpha_{2}$- Angular acceleration of the bottom pulley.

$$ {\text{Generally}},\quad J_{1} = \frac{{\pi \rho W\left( {r_{0}^{4} - r_{1}^{4} } \right)}}{2} + n_{sp} \left[ {\frac{{\rho V_{{2\left( { r_{3}^{2} + r_{4}^{2} } \right)}} }}{2}} \right] $$

Thus, the mass moment of Inertia of the head pulley (J_1_) was derived from Eq. (28) below:

28 $$ J_{1} = \frac{{\pi \rho W\left( {d_{0}^{4} - d_{1}^{4} } \right)}}{32} + n_{sp} \left[ {\frac{{\rho V_{2} \left( {d_{3}^{2} + d_{4}^{2} } \right)}}{8}} \right] $$

where n_sp_—Number of side plates of the head pulley.

Furthermore, the angular acceleration of the head pulley (${\upalpha }_{1}$) was determine from Eq. (29)

29 $$ \alpha_{1} = \frac{{2 \cdot V_{eb}^{2} }}{{d_{0} }} $$

Since the dimensions of the head and bottom pulleys are equal and of the same material, it therefore means: J_1_ $\alpha_{1}$ ≈ J_2_ $\alpha_{2}$^41,42^

#### Power (P)

The power required to operate this elevator was determined from Eq. (30) given as:

30 $$ P = \frac{{C \times Q \times S_{D} }}{367} $$

where C—Coefficient factor (for grain: 1.2)^43^.

In summary, the principal dimensions and mechanical parameters for the elevator system with respect to the pulley and belt are listed in Tables 1 and 2.

**Table 1:** Parameters for the shaft load (force) design.

Symbol	Description	Values
d_o_	External diameter of pulley	0.63 m
d_1_	Internal diameter of the pulley	0.61 m
W	Width of the pulley	0.44 m
t_1_	Thickness of the pulley plate	0.01 m
t_2_	Thickness of the side cover plate	0.01 m
d_3_	External diameter of pulley side cover plate	0.609998 m
d4	Internal diameter of pulley side cover plate	0.005 m
ρ	Mass density of pulley material	7879 kg/m^3^
g	Acceleration due to gravity	9.81 m/s^2^
D_d1_	Diameter of the head (drive) pulley	0.63 m
D_d2_	Diameter of the bottom (driven) pulley	0.63 m
S_D_	Centre to centre shaft distance	33.5 m
θ_1_	Angle of contact(wrap) for head pulleys	3.14 radians (≈ 180°)
θ_2_	Angle of contact(wrap) for bottom pulleys	3.14 radians (≈ 180°)
W_tB_	Width of the belt	0.4 m
t_B_	Thickness of belt	0.01 m
N_CL_	Number of cups per metre	6 cups/m
H_C_	height of cup	0.155 m
W_tC_	Width of the cup(projection)	0.23 m
P_C_ ≈ P_ec_	Perimeter of semi-ellipse(surface curve length of the cup	0.28 m
t_C_	Thickness of cup material(gauge)	0.0015 m
L_C_	Length of the cup	0.38 m
ρ_C_	Mass density of elevator cup material (AISI 1018)	7870 kg/m^3^
ρ_g_	Bulk density of grain(wheat)	795.3 kg/m^3^
Q	capacity of the bucket elevator	200 tons/h
n_sp_	Number of side cover plates of the head pulley	2
C	Coefficient factor for grain	1.2

**Table 2:** Calculated parameter values based on Table 1 definition.

Symbol	Description	Values
V_P_	Volume of pulley	1.44209 × 10^−2^ m^3^
W_P_	Weight of the head pulley	1113 N
L_B_	Length of belt	69 m
M_B_	Mass of belt	338.9 kg
ρ_B_	Density of belt	1228 kg/m^3^
W_B_	Weight of belt	3325 N
T_NC_	Total number of cups on the elevator	414
X	Hypotenuse of the triangle section	0.277 m
r	Radius of the semi-Ellipse section	0.0397 m
V_C_	Volume of cup material	2.31232033 × 10^−4^ m^3^
M_C_	Mass of cup	2 kg
W_C_	Weight of each cup	20 N
C_C_	Designed capacity of elevator cup	9.825627129 × 10^−3^ m^3^
M_W_	Maa of wheat per cup (excluding weight of cup)	5.23 kg
M_CW_	Mass of cup and wheat	7.23 kg
V_eb_	Velocity of belt	1.77 m/s
μ	Coefficient of friction	0.3
F_C_	Effective/circumferential force	6620 N
F_P_	Initial tension/preload force	7540 N
L_ce_	Length of belt contact on the pulley	0.99 m
N_CA_	Number of cups within the Arc length	6 cups
F_ce_	Centrifugal forces	88 N
F_1_	Tension on tight side of belt	10,938 N
F_2_	Tension on tight side of belt	4264 N
F_S_	Total force on shaft	16,315 N
α_1_,α_2_	Angular acceleration for head and bottom pulleys	9.946 rad/sec^2^
J_1_,J_2_	Mass moment of Inertia of head and bottom pulleys	107.09 kgm^2^
T	Total torque foe the shaft system	2316 Nm
P	Power Required	22 kw

The parameters from Table 1 were chosen at the conceptual design stage for the elevator system, the parameters in Table 2 were calculated using parameters in Table 1.

### Shaft diameters

**Figure 11:**
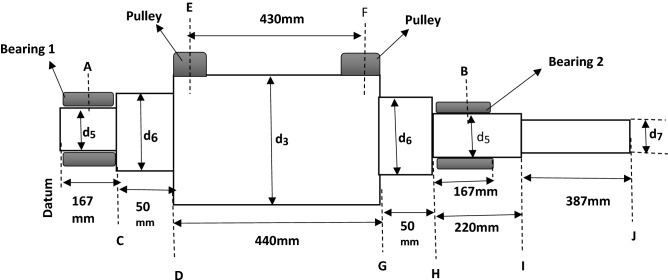
Schematic diagram of the shaft.

Considering the space on the elevator where the shaft will be mounted, a schematic diagram with length dimensions (mm) was developed as shown in Fig. 11.

Where d_5_, d_6_, d_3_, d_7_—diameter of each shoulder section of shaft in mm**.** Also, the length of the shaft cross sections is in mm.

The shaft design was based on determining the safe design diameters for each section of the shaft to carry loads efficiently without failure. Thus, the areas of the design analysis covered are:

### Support reactions

**Figure 12:**
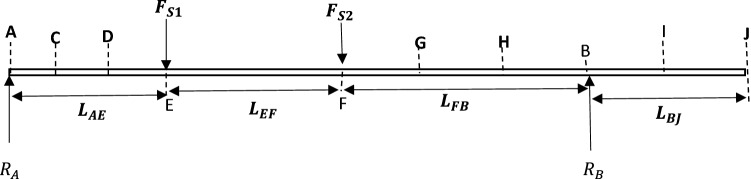
Free body diagram (FBD) of the shaft in beam representation.

This was determined from bending moment diagram. The free body diagram was first developed as from the schematic diagram in Fig. 11 by considering the shaft as a beam as shown in Fig. 12.

From the design, the two sections of the pulley side plates are fixed to the shaft. For this reason,

F_S1_ is equal to F_S2_ (i.e. F_S1_–F_S2_). Thus, F_S1_ is determined using Eq. (31)

31 $$ F_{S1} = \frac{{F_{S} }}{2} $$

According to Newton’s law for linear mechanical system, the sum of external forces acting on a rigid body is equal to the mass times the acceleration, as shown in Eq. (32)

32 $$ \Sigma F_{External} = Ma $$

D’ Alembert’s law also relates the sum of all forces acting on a rigid body as shown in Eq. (33)^44–46^:

33 $$ \Sigma F_{All} = 0 $$

Applying these two laws, the dynamic equation of motion for the shaft system are as follows in Eq. (34):

$$ \Sigma F_{All} = 0 $$

34 $$ \begin{gathered} R_{A} + R_{B} - F_{S1} - F_{S2} = 0 \hfill \\ R_{A} = F_{S1} + F_{S2} - R_{B} \hfill \\ \end{gathered} $$

Also, summation of all moments about Point A is equal to zero, as shown in Eqs. (35) and (36)

35 $$ \Sigma M_{A} = 0 $$

$$ R_{B} x \left( {L_{AE} + L_{EF} + L_{FB} } \right) - F_{S2} x \left( {L_{EF} + L_{AE} } \right) - F_{S1} \left( {L_{AE} } \right) = 0 $$

36 $$ R_{B} = \frac{{F_{S2} \times \left( {L_{EF} + L_{AE} } \right) + F_{S1} \left( {L_{AE} } \right) }}{{L_{AE} + L_{EF} + L_{FB} }} $$

where R_A_, R_B_ are the reactions from support bearings 1 and 2.

### Bending moment

**Figure 13:**
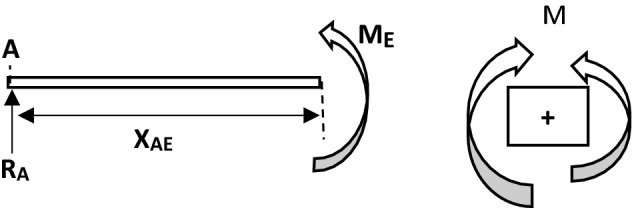
FBD for analysis of section AE.

Taking sections of the beam,

The bending moment at E from Fig. 13 was determined from Eq. (37),

Therefore, bending moment at E (M_E_), we have Eq. (37),

37 $$ M_{E} = R_{A} \; \times \; X_{AE} $$

Thus, the bending moment at A (i.e. X_AE_ = 0), is M_A_ = 0.

**Figure 14:**
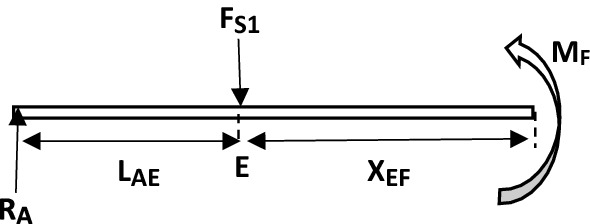
FBD for analysis of section EF.

The bending moment at F from Fig. 14 was determine from Eq. (38)

#### Bending moment at F (M_F_)

38 $$ M_{F} = R_{A} \left( {L_{AE} + X_{EF} } \right) - F_{S1} X_{EF} $$

Bending moment at G (M_G_) was determined from Eq. (39):

39 $$ M_{G} = R_{A} \left( {L_{AE} + L_{EF} + X_{FG} } \right) - F_{S1} \left( {L_{EF} + X_{FG} } \right) - F_{S2} X_{FG} $$

Bending moment at H (M_H_) was determined from Eq. (40):

40 $$ M_{H} = R_{A} \left( {L_{AE} + L_{EF} + X_{GH} } \right) - F_{S1} \left( {L_{EF} + L_{FH} } \right) - F_{S2} L_{FH} $$

Bending moment at B (M_B_) was determined from Eq. (41):

41 $$ M_{B} = R_{A} \left( {L_{AE} + L_{EF} + X_{FB} } \right) - F_{S1} \left( {L_{EF} + X_{FB} } \right) - F_{S2} X_{FB} $$

Bending moment at J (M_J_) was determined from Eq. (42):

42 $$ M_{X} = R_{A} \left( {L_{AE} + L_{EF} + L_{FB} + X_{BJ} } \right) + R_{A} X_{BJ} - F_{S1} \left( {L_{EF} + L_{FB} + X_{BJ} } \right) - F_{S2} \left( {L_{FB} + X_{BJ} } \right) $$

In summary, the principal and mechanical parameters of the elevator shaft system with respect to bending moment analysis are listed in Tables 3 and 4

**Table 3:** Parameters for bending moment analysis.

Symbol	Description	Values
F_S1_	Total force at section (point) E of the shaft	8157.5 N
F_S2_	Total force at section (point) F of the shaft	8157.5 N
L_AE_	Length from section (point) A to E of the shaft	138.5 mm
L_EF_	Length from section (point) E to F of the shaft	430 mm
L_FG_	Length from section (point) F to G of the shaft	5 mm
L_FH_	Length from section (point) F to H of the shaft	55 mm
L_FB_	Length from section (point) F to B of the shaft	138.5 mm
L_BJ_	Length from section (point) B to J of the shaft	523.5 mm

**Table 4:** Calculated parameter values based on Table 3 definitions.

Beam sections	X range	Bending moment (M_X_) at each points
A-E	At X_AE_ = 0	M_A_ = 0
	At X_AE_ = L_AE_	M_E_ = 1,129,814 Nmm
E–F	At X_EF_ = L_EF_	M_F_ = 1,129,814 Nmm
F–G	At X_FG_ = L_FG_	M_G_ = 1,089,026 Nmm
F–H	At X_FH_ = L_FH_	M_H_ = 681,151 Nmm
F–B	At X_FB_ = L_FB_	M_B_ = 0
B–J	At X_BJ_ = L_BJ_	M_J_ = 0

The parameters from Table 3 were chosen at the conceptual design stage for the elevator shaft, the parameters in Table 4 were calculated using parameters in Table 3.

**Figure 15:**
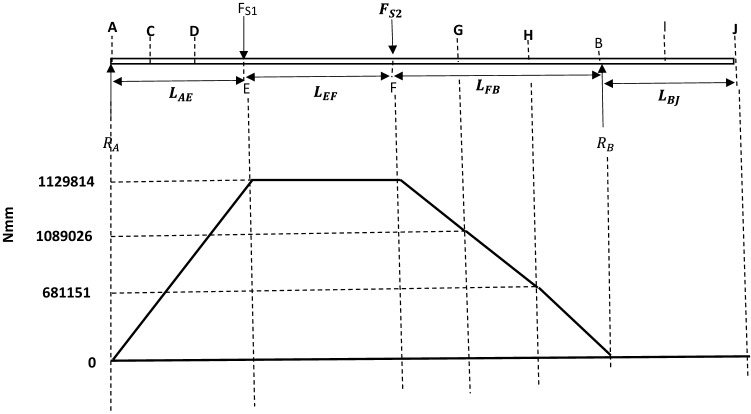
Bending moment diagram for the shaft analysis.

The bending moment diagram is shown in Fig. 15 below^47^.

Because stresses are usually higher at the points along the surface of the shaft where there are shoulders which invariably are stress risers, fatigue cracks are most likely to originate from these points and progress to fatigue failure of the shaft, hence for a safe design, the diameter of the shaft will be determined by considering this sections as follows:

#### For small diameter of the shoulder at H (d_5_)

At this section, torque and bending moments are present. This is also the section of the shaft where the bearings are installed, hence the section was designed with sharp shoulder (step). Assuming generous fillet radius, the standard 1^st^ estimated recommended for geometric stress concentration for shaft with shoulder fillet radius are:

$$ {\text{r}}/{\text{d }} = \, 0.0{2},{\text{ K}}_{{{\text{tB}}}} = { 2}.{7},{\text{ K}}_{{{\text{tT}}}} = {2}.{2} $$

where r—fillet radius, d—smaller diameter, K_tB_—theoretical stress concentration factor for bending, K_tT_—theoretical stress concentration factor for torsion.

Also, for quick conservative 1^st^ pass, we assume K_tT_ = K_FATT1_ = 2.7, K_tB_ = K_FATB1_ = 2.2

Where K_FATT_—Fatigue stress concentration factor for torsion in 1st estimate, K_FATB_—Fatigue stress concentration factor for bending in 1st estimate.

The diameter (d_5_) at the shoulder at H was determined using DE-Goodman criterion as shown in Eq. (43), considering alternating torque and midrange bending moment for rotating shaft was equal to zero.

43 $$ d_{5} = \left[ {\frac{{16n_{1} }}{\pi }\left\{ {\frac{{[4(K_{FATB1 } \; \times \;M_{H} )^{2} ]^{1/2} }}{{Se_{1} }} + \frac{{[3(K_{FATT1} \; \times \; Tm)^{2} ]^{1/2} }}{Sut}} \right\}} \right]^{{^{{{\raise0.7ex\hbox{$1$} \!\mathord{\left/ {\vphantom {1 3}}\right.\kern-\nulldelimiterspace} \!\lower0.7ex\hbox{$3$}}}} }} $$

where Se_1_—Endurance limit correction factor for 1st pass, Sut—Tensile strength of material (AISI 1045 steel, cold drawn), n_1_—Safety factor for 1st pass, M_H_—Alternating bending moment at H, Tm—midrange torque.

The endurance limit correction factor (Se_1_) for 1st pass was determined from Eq. (44) given as:

44 $$ Se_{1} = K_{a} \; \times \; K_{b1} \; \times \; K_{C} \; \times \; k_{d} \; \times \; K_{e} \; \times \; K_{F} \; \times \; Se^{\prime } $$

where K_a_—Surface factor, K_b1_—Size factor for 1st pass, K_C_—Load factor, K_d_—Temperature factor, K_e_—Reliability factor, K_F_—Miscellaneous factor, $Se^{\prime }$—Uncorrected endurance limit.

The surface factor (K_a_) was determined from Eq. (45) given as:

45 $$ K_{a} = aSut^{b} $$

where a and b are constant (Values of a = 4.51 and b = − 0.265 for machined/cold drawn shaft).

The size factor (K_b1_) = 1 (for 1st estimate), Load factor (Kc) = 1(for combined loading), temperature factor (K_d_) = 1(Assumed room temperature), Reliability factor (K_e_) = 1, Miscellaneous factor (K_f_) = 1.

The uncorrected endurance limit was determined from Eq. (46)

46 $$ Se^{\prime } = 0.5 Sut $$

Furthermore, considering that the stress type and material condition is uncertain, a safety factor (n_1_) of 5 (for the 1st pass) was used^48^

From Eq. (43), the standard shaft size (i.e. d_5_ = 90 mm) was analyzed to determine its fatigue and yielding factors of safety.

From DE-Goodman equation, the fatigue factor safety was determined from Eq. (47) given as:

47 $$ n_{5} = \frac{{Se_{5} \; \times \; Sut}}{{\left( {Sut \; \times \; \delta a_{5}^{\prime } + Se \; \times \; \delta m_{5}^{\prime } } \right)}} $$

where Se_5_—endurance limit correction factor for d_5,_ $\delta a_{5}^{1}$—Alternating von misses stress for d_5_, $\delta m_{5}^{1}$—midrange von misses stress for d_5._

The endurance limit correction factor for d_5_ (Se_5)_ was determined from Eq. (48) given as:

48 $$ Se_{5} = K_{a} \; \times \;K_{b5} \; \times \; K_{C} \; \times \; k_{d} \; \times \; K_{e} \; \times \; K_{F} \; \times \; Se^{\prime } $$

The size factor for d_5_ (K_b5_) was calculated from Eq. (49) given as:

49 $$ K_{b5} = 1.51\; \times \; d_{5}^{ - 0.157} $$

Also, the alternating von misses stress ($\delta a_{5}^{1}$) was determined from Eq. (50):

50 $$ \delta a_{5}^{1} = \frac{{K_{FATBH} \; \times \; 32M_{H} }}{{\pi d_{5}^{3} }} $$

K_FATBH_ is the fatigue stress concentration factor for bending at section(point) H and was determined using Eq. (51)

51 $$ K_{FATBH} = 1 + \frac{{\left( {K_{tBH} - 1} \right)\sqrt {r_{H} } }}{{\left( {\sqrt {r_{H} } + \sqrt {a_{B} } } \right)}} $$

where $\sqrt {a_{B} }$—is the Neuber constant for bending given in Eq. (52) as:

52 $$ \sqrt {a_{B} } = 0.246 - 3.08 \; \times \; 10^{ - 3} Sut + 1.51 \; \times \; 10^{ - 5} Sut^{2} - 2.67\; \times \; 10^{ - 8} Sut^{3} $$

$r_{H}$ is the fillet radius at H, K_tBH_ is the theoretical stress concentration factor for bending at point H and its value was determine from chart of theoretical stress concentration for shaft with shoulder fillet in bending for $\frac{{d_{6} }}{{d_{5} }}$ against $\frac{{r_{H} }}{{d_{5} }}$^49^.

d_6_ was determined from Eq. (53):

53 $$ d_{6} = d_{5} + 2 x h_{SH} $$

where $h_{SH}$ is the shoulder height at section H.

Considering the bearing(self-aligning bearing with adaptive sleeve) placement at section H(d_5_) and the standard shaft size (d_5_ = 90 mm), the fillet radius and shoulder height for the shaft from bearing catalogue are $r_{H} = 2\;{\text{mm}}\; and\; h_{SH} = 4\;{\text{mm}}$^50^.

Similarly, the midrange von misses stress for d_5_ was determined from Eq. (54)

54 $$ \delta m_{5}^{^{\prime}} = \left[ {3\left( {\frac{{K_{FATTH } \times \;16Tm}}{{\pi d_{5}^{3} }}} \right)^{2} } \right]^{{{\raise0.7ex\hbox{$1$} \!\mathord{\left/ {\vphantom {1 2}}\right.\kern-\nulldelimiterspace} \!\lower0.7ex\hbox{$2$}}}} $$

K_FATTH_ is the fatigue stress concentration factor for torsion at section H(d_5_) and was determined from Eq. (55):

55 $$ K_{FATTH} = 1 + \frac{{\left( {K_{tTH} - 1} \right)\sqrt {r_{H} } }}{{(\sqrt {r_{H} } + \sqrt {a_{T} } }} $$

where $\sqrt {a_{T} }$—is the Neuber constant for torsion given in Eq. (56) as:

56 $$ \sqrt {a_{T} } = 0.190 - 2.57\; \times \; 10^{ - 3} Sut\; + \;1.35\; \times \; 10^{ - 5} Sut^{2} \; - \;2.62\; \times \; 10^{ - 8} Sut^{3} $$

K_tTH_ is the theoretical stress concentration factor for torsion at point H and its value was determine from chart of theoretical stress concentration for shaft with shoulder fillet in torsion for $\frac{{d_{6} }}{{d_{5} }}$ against $\frac{{r_{H} }}{{d_{5} }}$^49^.

Furthermore, the yielding factor of safety for section H (d_5_) was determined from Eq. (57):

57 $$ n_{y5} = \frac{Sy}{{\delta max_{5}^{^{\prime}} }} $$

where Sy—yield strength of AISI steel material, $n_{y5}$—yielding factor of safety for section H, $\delta max_{5}^{^{\prime}}$—Von misses Maximum stress at section H (d_5_) determined from Eq. (58)

58 $$ \delta max_{5}^{^{\prime}} = \left[ {\left( {\frac{{K_{FATBH } x32M_{H} }}{{\pi d_{5}^{3} }}} \right)^{2} + 3\left( {\frac{{K_{FATTH } x 16Tm}}{{\pi d_{5}^{3} }}} \right)^{2} } \right]^{{{\raise0.7ex\hbox{$1$} \!\mathord{\left/ {\vphantom {1 2}}\right.\kern-\nulldelimiterspace} \!\lower0.7ex\hbox{$2$}}}} $$

Generally, for safe design, $\delta max_{5}^{^{\prime}}$ ≤ Sy^51^.

#### For small diameter of the shoulder at G (d_6_)

At this section, there was presence of bending and torsion***. ***The fatigue factor of safety was determined from Eq. (59)

59 $$ n_{6} = \frac{{Se_{6} x Sut}}{{\left( {Sut x \delta a_{6}^{^{\prime}} + Se x \delta m_{6}^{^{\prime}} } \right)}} $$

where Se_6_—endurance limit correction factor for d_6,_ $\delta a_{6}^{1}$–Alternating von misses stress for d_6_, $\delta m_{6}^{1}$- midrange von misses stress for d_6._

The endurance limit correction factor for d_6_ (Se_6_) was determined from Eq. (60) given as:

60 $$ Se_{6} = K_{a} \; \times \;K_{b6} \; \times \; K_{C} \; \times \; k_{d} \; \times \;K_{e} \; \times \; K_{F} \; \times \; Se^{^{\prime}} $$

The size factor for d_6_ (K_b6_) was calculated from Eq. (61) given as:

61 $$ K_{b6} = 1.51 \; \times \;d_{6}^{ - 0.157} $$

Also, the alternating von misses stress ($\delta a_{6}^{1}$) was determined from Eq. (62):

62 $$ \delta a_{6}^{1} = \frac{{K_{FATBG} \; \times \; 32M_{G} }}{{\pi d_{6}^{3} }} $$

K_FATBG_ is the fatigue stress concentration factor for bending at section(point) G and was determined using Eq. (63):

63 $$ K_{FATBG} = 1 + \frac{{\left( {K_{tBG} - 1} \right)\sqrt {r_{G} } }}{{(\sqrt {r_{G} } + \sqrt {a_{B} } }} $$

where $r_{G}$ is the fillet radius at G, K_tBG_ is the theoretical stress concentration factor for bending at point G and its value was determined from chart of theoretical stress concentration for shaft with shoulder fillet in bending for $\frac{{d_{3} }}{{d_{6} }}$ against $\frac{{r_{G} }}{{d_{6} }}$^49^

In design of machine shaft where bending and torsion loading are present, small value of D/d near 1 is recommended. For this, d_3_ was determined from Eq. (64)

64 $$ d_{3} = 1.1 \; \times \; d_{6} $$

To obtain least stress concentration factor for shaft shoulder, the fillet height was assumed to be equal to the fillet radius^52^ Thus, r_G_ was obtained from Eq. (65):

65 $$ r_{G} = \frac{{d_{3} - d_{6} }}{2} $$

Similarly, the midrange von misses stress for d_6_ was determined from Eq. (66)

66 $$ \delta m_{6}^{\prime } = \left[ {3\left( {\frac{{K_{FATTG } \; \times \; 16Tm}}{{\pi d_{6}^{3} }}} \right)^{2} } \right]^{{{\raise0.7ex\hbox{$1$} \!\mathord{\left/ {\vphantom {1 2}}\right.\kern-\nulldelimiterspace} \!\lower0.7ex\hbox{$2$}}}} $$

K_FATTG_ is the fatigue stress concentration factor for torsion at section G(d_6_) and was determined from Eq. (67):

67 $$ K_{FATTG} = 1 + \frac{{\left( {K_{tTG} - 1} \right)\sqrt {r_{G} } }}{{\left( {\sqrt {r_{G} } + \sqrt {a_{T} } } \right)}} $$

K_tTG_ is the theoretical stress concentration factor for torsion at point G and its value was determined from chart of theoretical stress concentration for shaft with shoulder fillet in torsion for $\frac{{d_{3} }}{{d_{6} }}$ against $\frac{{r_{G} }}{{d_{6} }}$^49^.

Furthermore, the yielding factor of safety for section G (d_6_) was determined from Eq. (68):

68 $$ n_{y6} = \frac{Sy}{{\delta max_{6}^{\prime } }} $$

where $n_{y6}$—yielding factor of safety for section G, $\delta max_{6}^{\prime }$—Von Misses Maximum stress at section G (d_6_), it was determined from Eq. (69)

69 $$ \delta max_{6}^{\prime } = \left[ {\left( {\frac{{K_{FATBG } \times 32M_{G} }}{{\pi d_{6}^{3} }}} \right)^{2} + 3\left( {\frac{{K_{FATTG } \times 16Tm}}{{\pi d_{6}^{3} }}} \right)^{2} } \right]^{{{\raise0.7ex\hbox{$1$} \!\mathord{\left/ {\vphantom {1 2}}\right.\kern-\nulldelimiterspace} \!\lower0.7ex\hbox{$2$}}}} $$

For safe design, $\delta max_{6}^{\prime }$ ≤ Sy.

#### For small diameter of the shoulder at I (d_7_)

At this section, only torque is present. The load is entirely shear stress; hence the yielding factor of safety was determined from Eq. (70):

70 $$ n_{y7} = \frac{Sys}{{\tau max_{7}^{\prime } }} = \frac{0.577 \; \times \; Sy}{{\tau max_{7}^{\prime } }} $$

where Sys—yield shear strength, $\tau max_{7}$—maximum shear stress at I (d7) and $\delta max_{7}^{\prime }$—Von misses Maximum stress at section I (d_7_) where determined from Eqs. (71) and (72):

71 $$ \tau max_{7} = \frac{{\delta max_{7}^{\prime } }}{\sqrt 3 } $$

72 $$ \delta max_{7}^{\prime } = \sqrt 3 x \left( {\frac{{K_{FATTI} \; \times \; 16Tm}}{{\pi d_{7}^{3} }}} \right) $$

where K_FATTI_ is the fatigue stress concentration factor for torsion at section I (d_7_) and was determined from Eq. (73):

73 $$ K_{FATTI} = 1 + \frac{{\left( {K_{tTI} - 1} \right)\sqrt {r_{I} } }}{{\left( {\sqrt {r_{I} } + \sqrt {a_{T} } } \right)}} $$

K_tTI_ is the theoretical stress concentration factor for torsion at point I and its value was determined from chart of theoretical stress concentration for shaft with shoulder fillet in torsion for $\frac{{d_{5} }}{{d_{7} }}$ against $\frac{{r_{I} }}{{d_{7} }}$^49^.

Also, r_I_ is the fillet radius at I and was determined from Eq. (74):

74 $$ r_{I} = \frac{{d_{5} - d_{7} }}{2} $$

The value of d_7_ was determined from Eq. (75):

75 $$ d_{7} = \frac{{d_{5} }}{1.1} $$

Thus, for safe design, $\tau max_{7}^{\prime }$ $<$ $Sys$.

In summary, the principal parameters for determining the safe diameters of the sections of the elevator shaft are listed in Tables 5 and 6

**Table 5:** Parameter for shaft diameter.

Symbol	Description	Value
n_1_	Fatigue factor of safety for 1st pass	5
Sut	Tensile strength fot AISI 1045 steel	625 N/mm^2^≈ 0.625 Gpa
Sy	yield strength fot AISI 1045 steel	530 N/mm^2^

**Table 6:** Calculated parameter values based on Table 5 definition.

Symbol	Description	Value
Ka	Surface factor	0.819
Se'	Uncorrected endurance limit	312.50 N/mm^2^
Se_1_	Endurance limit correction factor for 1st pass	256 N/mm^2^
d_5_	Small diameter at shoulder H	90 mm
n_5_	Fatigue factor of safety at d_5_	6
Se_5_	Endurance limit correction factor for d_5_	191 N/mm^2^
K_b5_	Size factor for d_5_	0.745
δa_5_'	Alternating von misses stress at d_5_	19.788 N/mm^2^
K_FATBH_	Fatigue stress concentration factor for bending at section H	2.08
$\sqrt {a_{B} }$	Neuber constant for bending	0.244
K_tBH_	Theoretical stress concentration factor for bending at section H	2.27
r_H_	Fillet radius at H	2 mm
h_SH_	Shoulder height at section H	4 mm
d_6_	Small diameter at shoulder G	98 mm
δm_5_'	Midrange von misses stress at d_5_	39.779 N/mm^2^
K_FATTH_ $\sqrt {a_{B} }$	Fatigue stress concentration factor for torsion at section H	1.42
K_tTH_	Theoretical stress concentration factor for torsion at section H	1.48
$\sqrt {a_{T} }$	Neuber constant for torsion	0.188
n_y5_	Yielding fatigue factor of safety for d_5_	12
δmax_5_'	Von misses maximum stress at d_5_	44.43 N/mm^2^
n_6_	Fatigue factor of safety at d_6_	7
Se_6_	Endurance limit correction factor for d_6_	188 N/mm^2^
δa_6_'	Alternating von misses stress at d_6_	18.64 N/mm^2^
K_b6_	Size factor for d_6_	0.7351
K_FATBG_	Fatigue stress concentration factor for bending at section G	1.58
K_tBG_	Theoretical stress concentration factor for bending at section G	1.64
r_G_	Fillet radius at G	6 mm
d_3_	Large diameter at G	110 mm
δm_6_'	Midrange von misses stress at d_6_	26.688 N/mm^2^
K_FATTG_	Fatigue stress concentration factor for torsion at G	1.23
K_tTG_	Theoretical stress concentration factor for torsion at section G	1.25
n_y6_	Yielding fatigue factor of safety for d_6_	16
δmax_6_'	Von misses maximum stress at d_6_	32.54 N/mm^2^
d_7_	Small diameter at shoulder I	82 mm
r_I_	Fillet radius at I	4 mm
K_tTI_	Theoretical stress concentration factor for torsion at section I	1.34
K_FATTI_	Fatigue stress concentration factor for torsion at I	1.31
δmax_7_'	Von misses maximum stress at d_7_	48.52 N/mm^2^
τmax_7_	maximum shear stress at I	28.01 N/mm^2^
Sys	Yield shear strength	305.81 N/mm^2^
n_y7_	Yielding fatigue factor of safety for d_7_	11

The parameters from Table 5 were chosen at the conceptual design stage for the elevator shaft, the parameters in Table 6 was calculated using parameters in Table 5.

#### Model development

**Figure 16:**
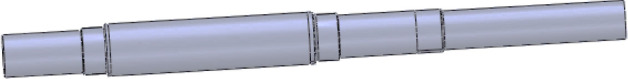
Shaft model.

The model of the shaft was produced using Solidworks, which is a solid modelling computer-aided design and computer aided engineering programme. The 2D line sketch of the half section of the shaft with reference to a center axis line was first created. The dimensions from the design calculations were then added to the sketch so as to define its sizes. The revolved boss/base tool of the feature manager was then used to rotate the contour of the line sketch about the axis line thereby creating a round shape object which is the shaft model as shown in Fig. 16.

#### Simulation and boundary conditions

Because the shaft will be subjected to static and cyclic loading over its service life, most commonly, the shaft will tend to fail under fatigue. For proper design of the shaft, a stress-life (S–N) curve which is one of the fatigue life methods to predict the number of cycles to failure for specific load level the shaft will be subjected to was constructed. A well-defined stress-life (S–N) characteristic of the shaft material will aid in obtaining precise fatigue life prediction for the shaft. The simulation analysis of the shaft model for both static and fatigue loading was done using Solidworks engineering software. The software uses finite element analysis (FEA) in predicting real world physical behavior of modeled mechanical components^53^. For this analysis, the shaft model was first created from the calculated shaft sizes, followed by static analysis using the following calculated parameters in Table 7.

**Table 7:** Parameters for static analysis.

Symbol	Description	Values
F_S1_	Total force at section (point) E of the shaft	8157.5 N
F_S2_	Total force at section (point) F of the shaft	8157.5 N
T	Total torque foe the shaft system	2316 Nm

The step-by-step procedure for the simulation on solidworks are as follows:

#### Material

AISI 1045 cold drawn steel was selected as the shaft material. The material properties of the shaft material are given in Table 8.

**Table 8:** Properties of the shaft material.

Property	Value
Elastic modulus	2.05 × 10^11^ N/m^2^
Poison’s ratio	0.29
Shear modulus	8 × 10^10^ N/m^2^
Mass density	7850 kg/m^3^
Tensile strength	6.25 × 10^8^ N/m^2^
Yield strength	5.30 × 10^8^ N/m^2^

#### Fixtures

**Figure 17:**
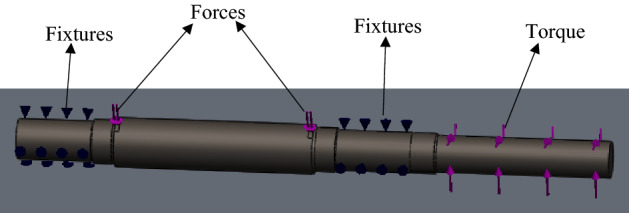
Fixtures and external loads on the shaft model.

The shaft has a bearing on the left and right hand of the shaft, for this reason, bearing fixture was applied at the left and right hand end of the shaft as shown in Fig. 17.

#### External loads

Equal forces of 8157.5 N were applied at the slit sections of the shaft where the pulley is fixed. A torque of 2316 Nm was also applied to the shaft end at a position the gear drive motor will be coupled, as also shown in Fig. 17.

#### Mesh

**Figure 18:**
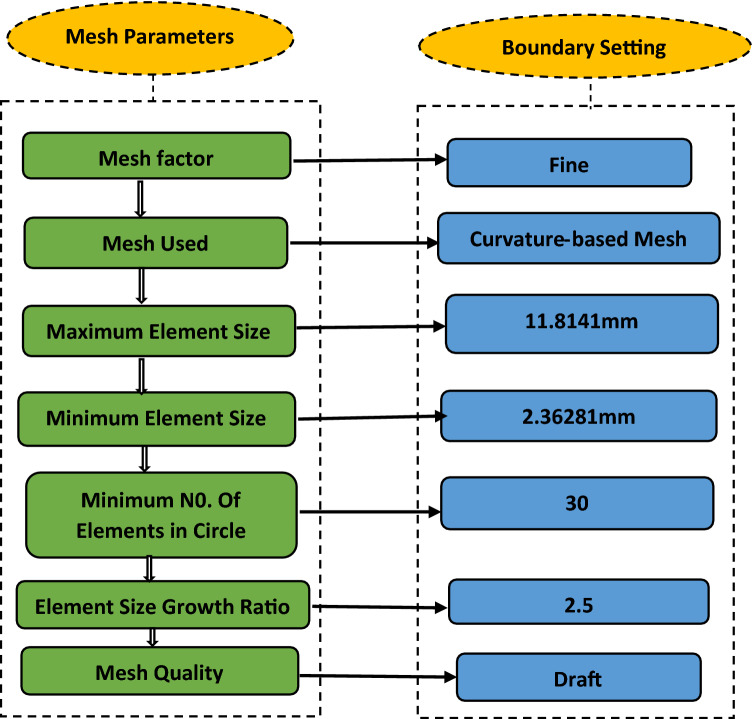
Mesh setting sequence.

This is a crucial step in design analysis which involves subdividing the model into small pieces. A good mesh increases the accuracy of the simulation result. Mesh setting was first carried out before the meshed model of the shaft was produced. The mesh setting was done in steps as shown in Fig. 18.

**Figure 19:**
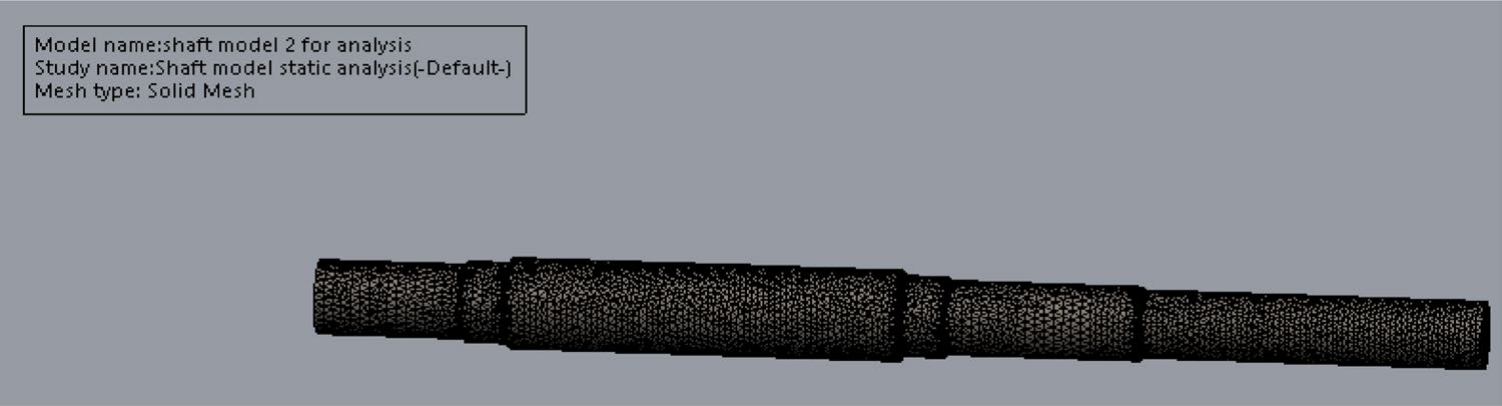
Meshed model of the head shaft.

The meshed model and a complete mesh information as shown in Fig. 19 and Table 9 were subsequently generated after the application of the parameter setting. The simulation was then put to run after the setting process.

**Table 9:** Mesh generated details.

Parameters	Details
Study name	Shaft model Satic Analysis
Mesh type	Solid Mesh
Mesh used	Curvature-Based Mesh
Jacobian points	4 Points
Maximum element size	11.8141 mm
Minimum element Size	2.36281
Mesh quality	Draft
Total nodes	21,903
Total elements	106,936
Maximum aspect ratio	7.3867
% of elements with aspect ratio < 3	98.3
% of elements with aspect ratio < 10	0
Time to complete mesh (hh:mm:ss)	00:00:15

The parameter setting used for fatigue are as follows.

- *Criterion* MAX Von Misses Stress
- *Computing alternating stress* Equivalent stress (Von misses)
- *Mean stress correction* Goodman

The simulation results for the maximum von misses stress and that of the yielding factor of safety were determined from the static analysis. Following that, fatigue analysis was carried out with the same software and the value for the fatigue life, fatigue load and S–N curve were equally obtained from the simulation results.

## Results and discussion

**Figure 20:**
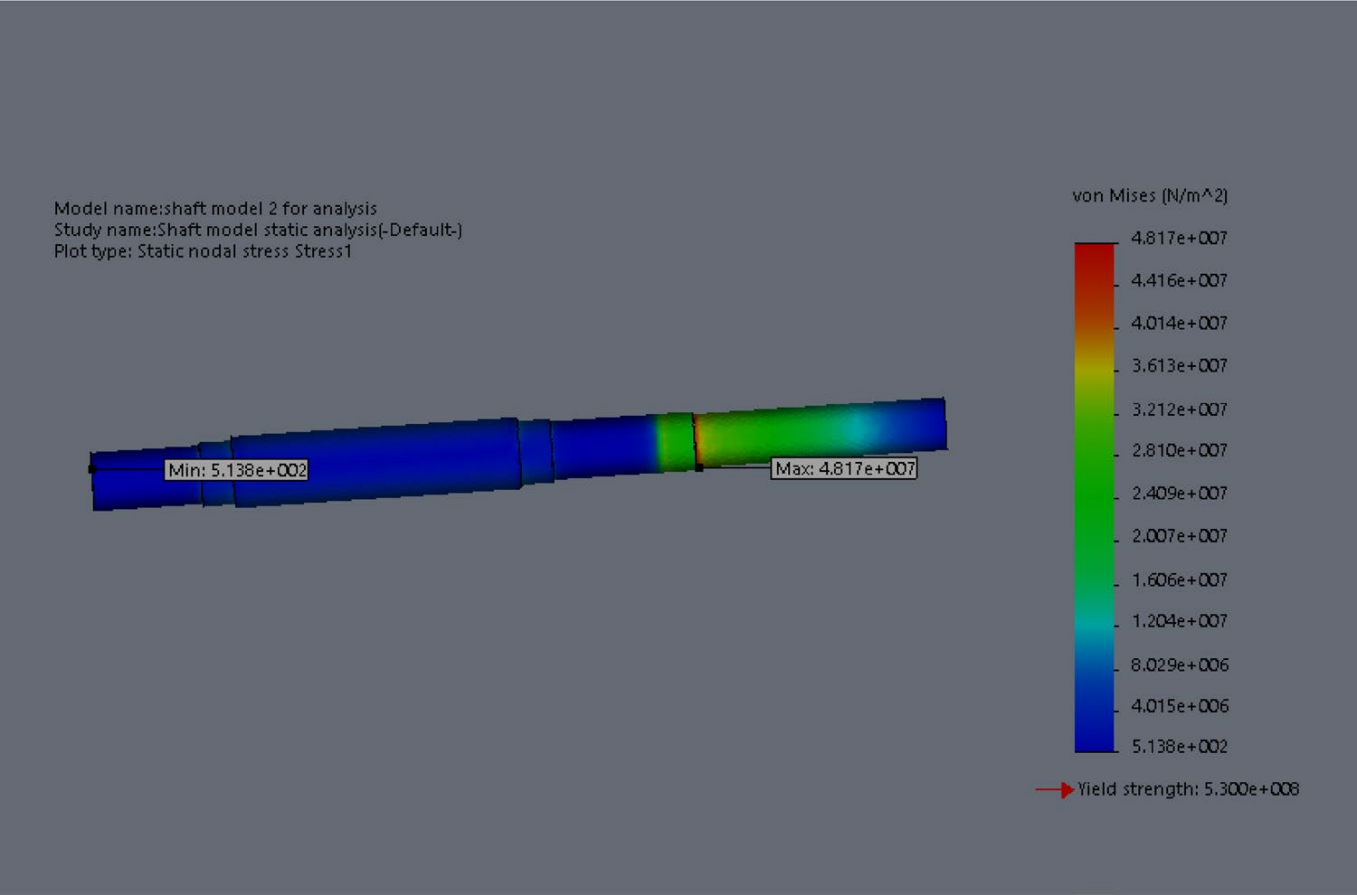
Plots of the stress.

**Figure 21:**
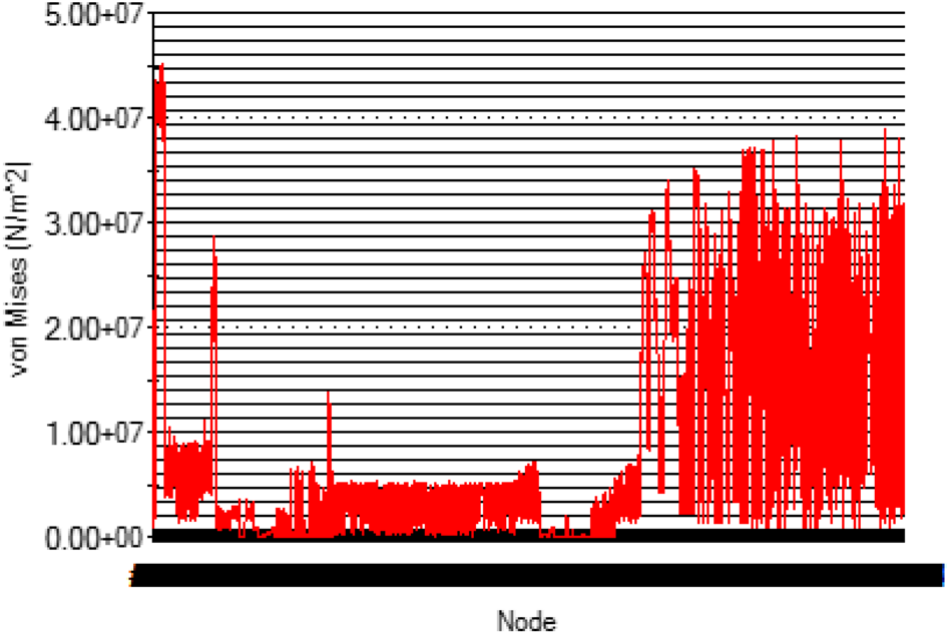
Simulation graph for stress.

**Figure 22:**
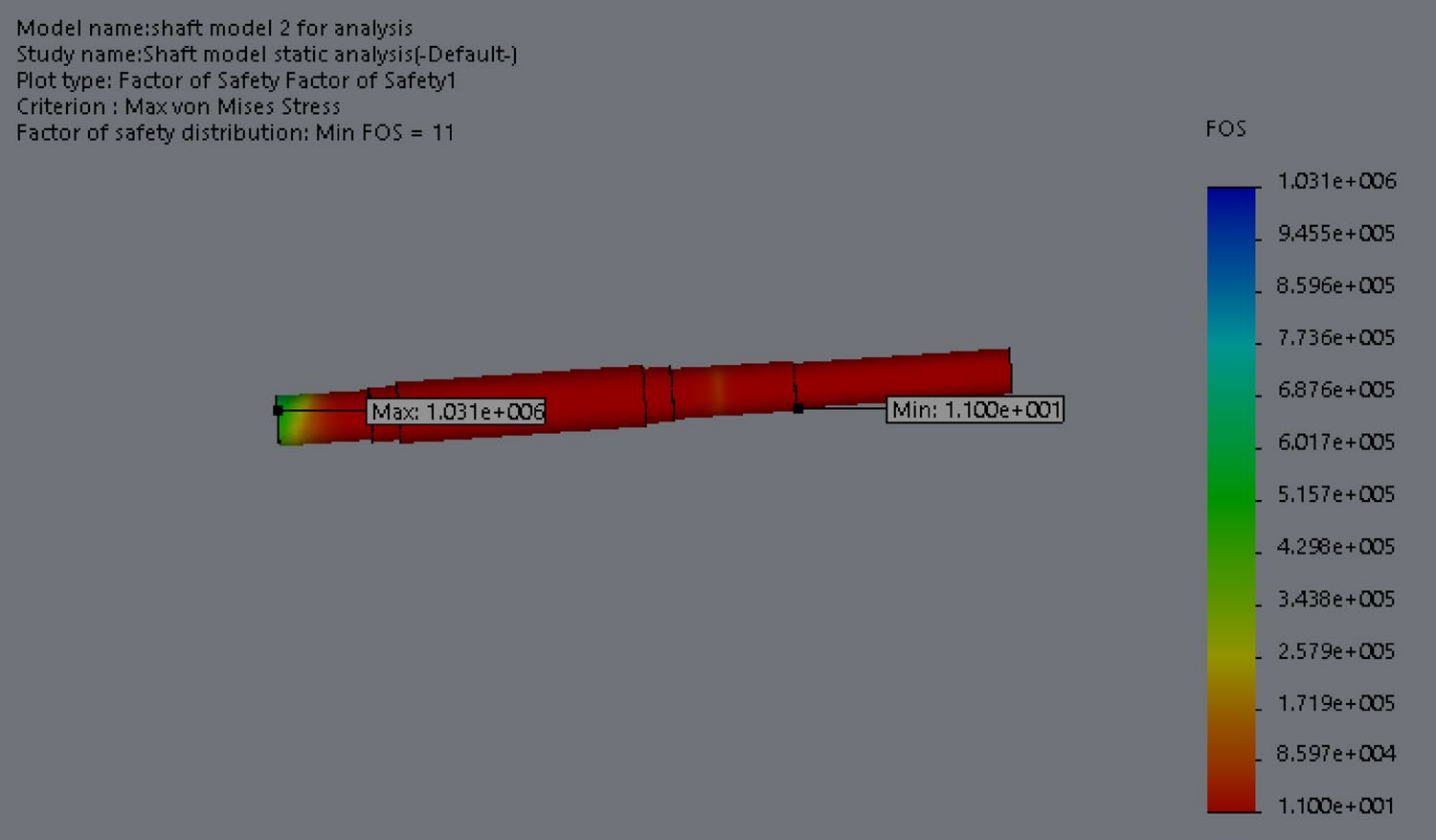
Plots of factor of safety.

**Figure 23:**
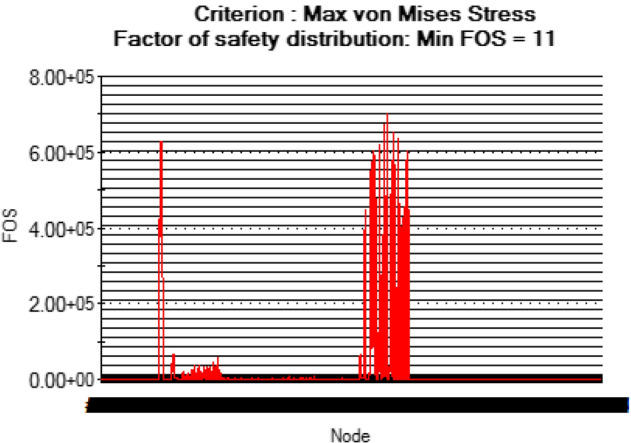
Simulation graph for factor of safety.

**Figure 24:**
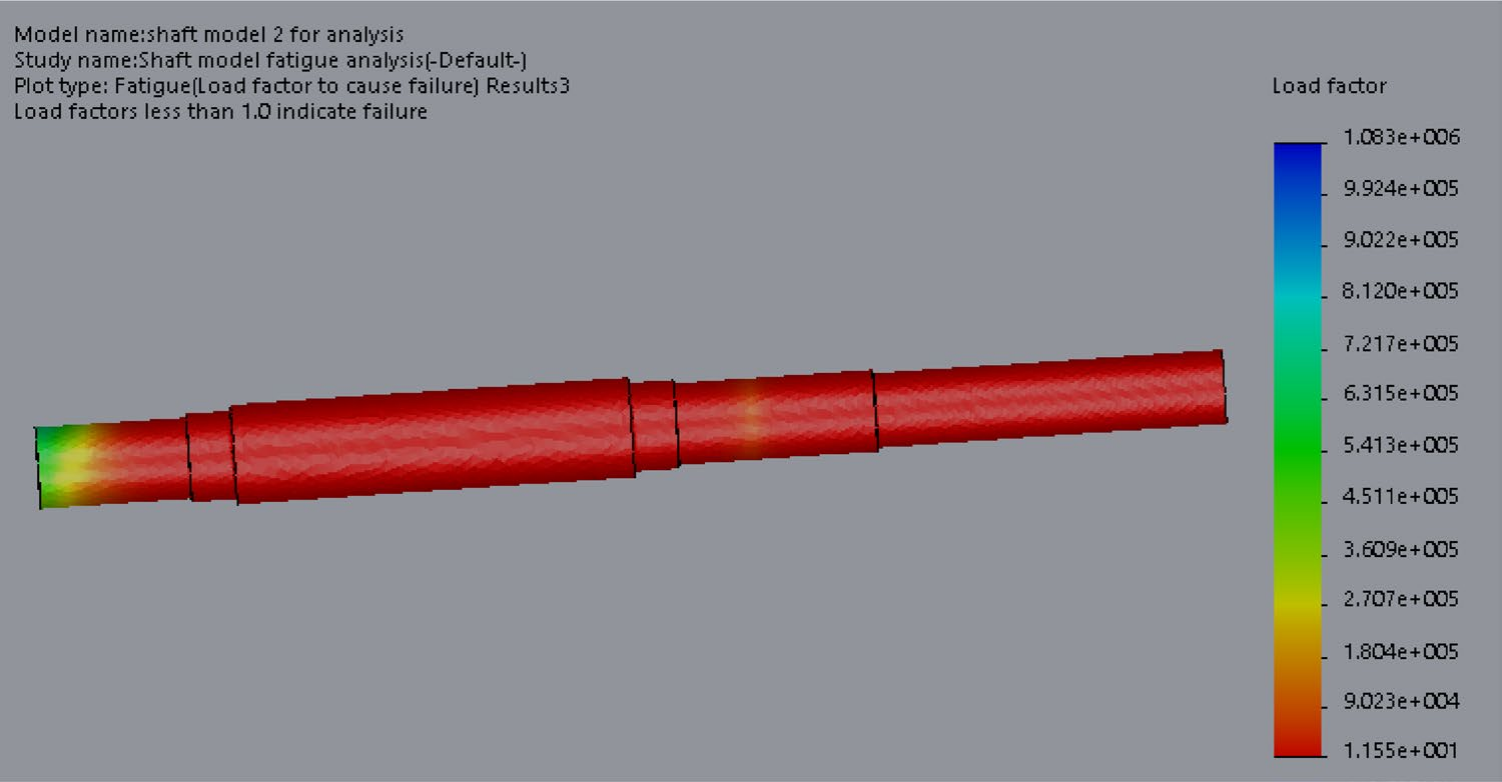
Fatigue load plot.

**Figure 25:**
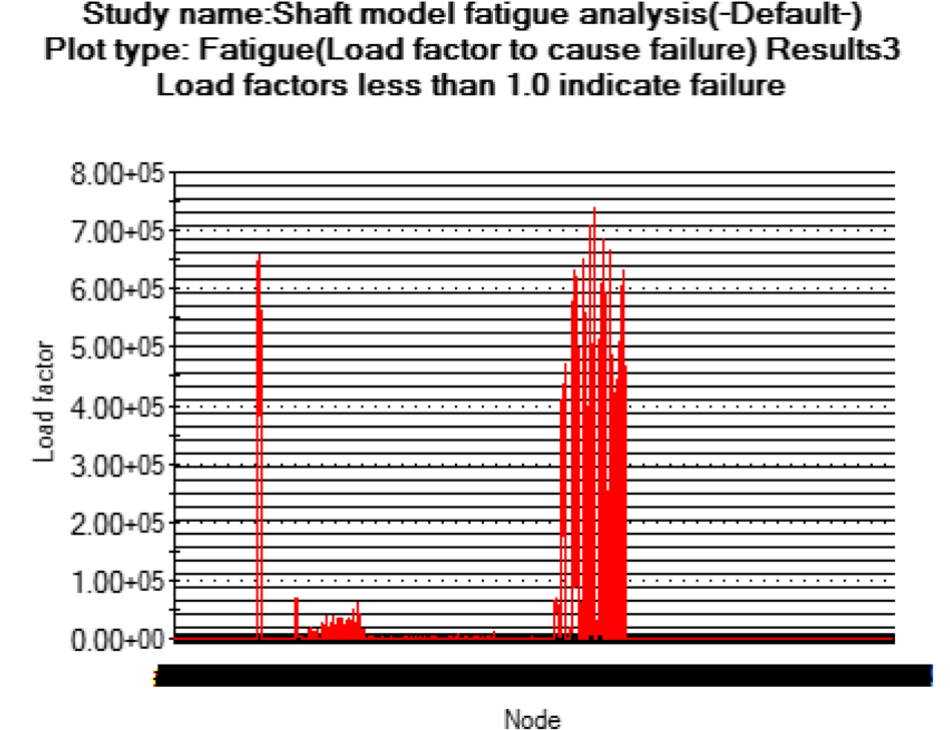
Simulation graph for fatigue load.

**Figure 26:**
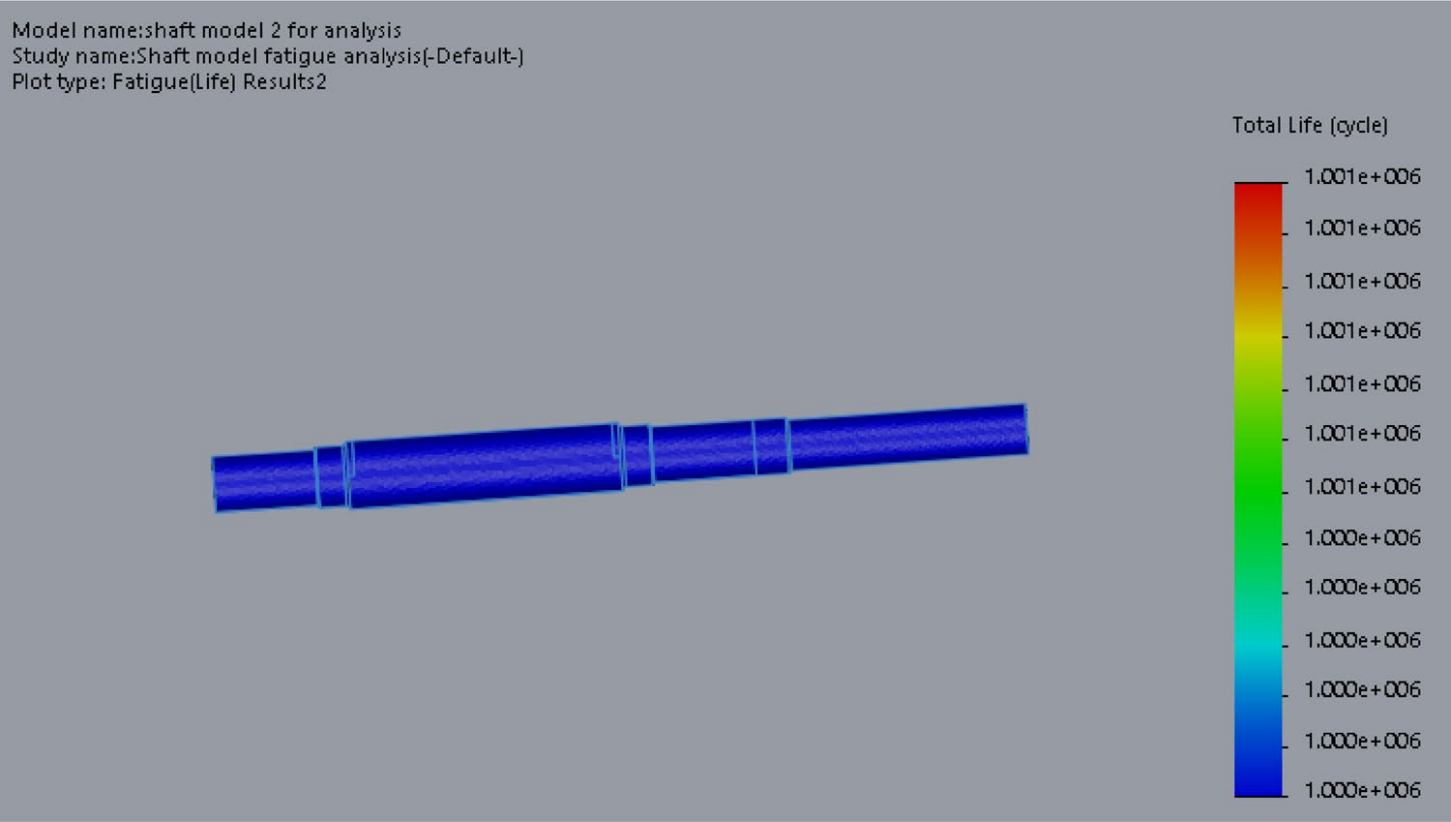
Fatigue life plot.

**Figure 27:**
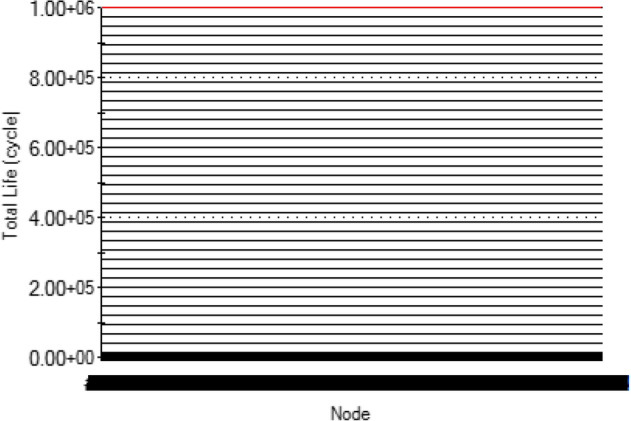
Simulation graph for fatigue life.

**Figure 28:**
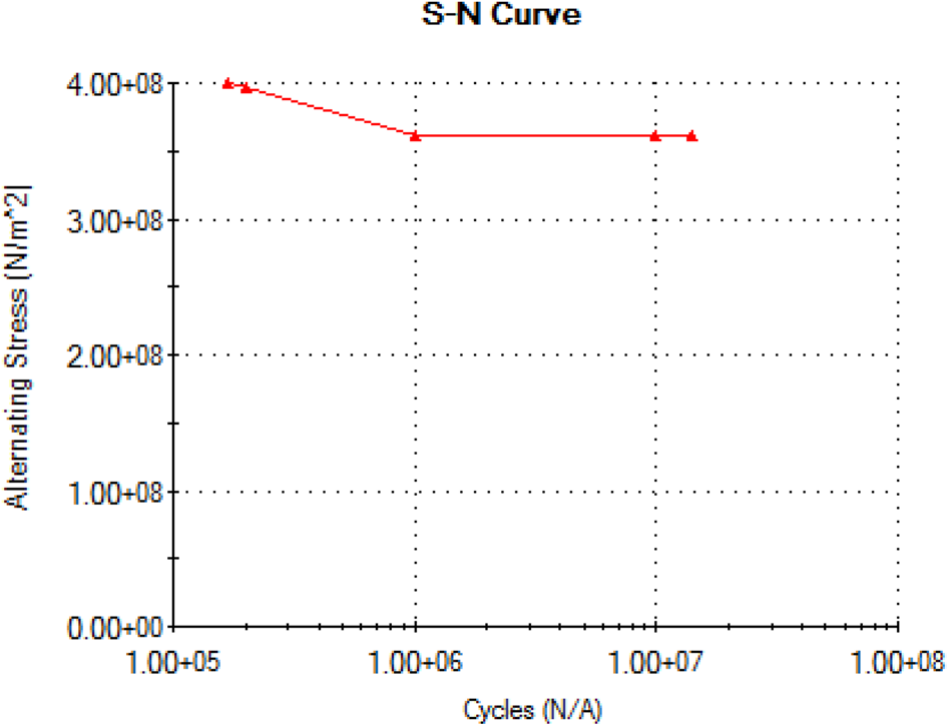
Fatigue stress-life (S–N) curve.

At this conceptual design stage, the performance of the head shaft was tested and compared with value obtained from the analytic design calculations. The results (Figs. 20, 21, 22, 23, 24, 25, 26, 27, 28) were obtained from the simulation analysis of the model. The results from static analysis are Figs. 20, 21, 22 and 23 while that of fatigue analysis are Figs. 24, 25, 26, 27 and 28.

### Plots from static analysis

See Figs. 20, 21, 22 and 23.

### Plots from fatigue analysis

From the plots (Figs. 20 and 21), the maximum Von misses stress occurred at the small diameter of section I and the value was 4.817 × 10^7^ N/m^2^ which was equivalent to the value (4.852 × 10^7^ ≈ 48.52 N/mm^2^) obtained from the design calculation. The value of the maximum von misses stress was less than the yield strength value of the shaft material, this signifies that the shaft will operate in the elastic region, this means that the designed diameters calculated for the shaft are safe for the elevator system operation. From the plots in Figs. 22 and 23, the yielding factor of safety at the small diameter section of I was 11 and was equivalent to the calculate value ($n_{y7}$ = 11), this shows that a generous design factor was considered for the head shaft design. The plots in Figs. 24 and 25 show that the load factor was 11.55, this was higher than 1 (i.e. 11.55 > 1), hence this signifies the shaft will not go into failure mode within the 1.0 × 10^6^ cycles. The plots of Figs. 26 and 27 show that the value of the fatigue life was 1.0 × 10^6^ cycles, this result signifies infinite life for the shaft. This was validated from the result of the S–N curve of Fig. 28 which shows a fatigue strength of 3.62 × 10^8^ N/m^2^, this value was higher than the value of the maximum von misses stress (4.817 × 10^7^ N/m^2^), thus signifying that the shaft will not fail but will survive indefinitely in operation. The closeness of the calculated values with the values from the analysis show that FEA is a very useful and effective tool for design of mechanical components that will be subjected to combined loading as well as for predicting the service life of the component.

Also, this research used the DE-Goodman criterion because of the need for accurate prediction of failure for the ductile shaft material and also due to the combined load of bending and torsion on the head shaft. The analysis also showed the difficulty of getting optimized numerical values for some design parameters such as the material variation and shaft weight, this point may surely be future research work for a more accurate and optimized head shaft model analysis. Also, fatigue test of the developed model should be performed in future research to confirm the theoretical data.

## Conclusion

This study presented a detailed approach for the design, modeling and simulation analysis of the head shaft of a belt bucket elevator for transporting grains (wheat). As a critical component of the elevator system, the forces acting on the shaft were first determined, the load type and the stresses at potential critical locations were considered in the design calculations. To ensure a proper design, modeling and simulation analysis were performed on the designed shaft so as to determine the fatigue strength and to make accurate prediction of the fatigue life of the head shaft. The result from the simulation analysis shows that the designed diameters for the shaft were safe for operation considering the 200 tons/h design capacity of this bucket elevator system.

This study can be used in industry as a reference for predicting the service life of shaft designed for different capacity of elevator systems. It will also help industrial engineers to have better understanding of the behavior of elevator shaft as well as to gain advanced knowledge on how to improve the fatigue life of shafts in manufacturing machines using FEA tools; this will directly reduce cost associated to downtime.

Note also that the complexity of the model is such that authors where not able to show and discuss all the details of their work. The authors stay away with pleasure to the disposal of the interested readers for any further discussion on the approach followed here.

## Data Availability

All data relevant to the study are included in the article. In addition, the datasets generated during and/or analyzed during the current study are available from the corresponding author on reasonable request.
